# Bar press durations as a reliable and robust measure of frustration-related operant behavior: Sensitivity to incentive downshift and dose-response paradigms

**DOI:** 10.1371/journal.pone.0296090

**Published:** 2023-12-21

**Authors:** Yorkiris Mármol Contreras, Tileena E. S. Vasquez, Poonam Shah, Kelsey Payne, Jessica Di Re, Fernanda Laezza, Thomas A. Green

**Affiliations:** 1 Department of Pharmacology and Toxicology, The University of Texas Medical Branch, Galveston, Texas, United States of America; 2 Center for Addiction Sciences and Therapeutics, The University of Texas Medical Branch, Galveston, Texas, United States of America; 3 Mental Health Research Group, The University of Texas Medical Branch, Galveston, Texas, United States of America; University of Arizona College of Medicine, UNITED STATES

## Abstract

In humans, frustrating experiences are known to trigger relapse events and individuals with higher frustration intolerance show increased risk of developing substance use disorders (SUDs). Despite this clear relationship, frustration-related behavior is seldom studied concurrently with self-administration behavior in rodent models. A major obstacle has been the lack of robust, quantitative assays of frustration-related operant behavior thus far. In previous work, we identified increased bar press (BP) durations in response to frustrating conditions in rats self-administering natural or drug rewards. Here, to propose BP durations as a measure of frustration-related behavior, we conducted an operant successive negative contrast (oSNC) study and found that increases in BP durations are observed in the absence of increased effort, providing evidence that this is a psychological phenomenon. Moreover, we assess the viability of widespread use of BP duration measurements as a behavioral tool by quantifying performance as it pertains to sensitivity, robustness, replicability, and sex differences. We conclude that increases in BP durations are a highly sensitive psychological response to frustrating conditions and that this measure is robust, replicable, and applicable to both sexes.

## Introduction

In addition to craving, impulsivity, and habit, frustration is a major facet of substance use disorders (SUDs). Individuals with greater frustration-intolerance are at a higher risk of developing SUDs [1], and have more relapses [2]. Coping with frustration or anger has been shown to underlie 20–50% of relapses associated with negative intrapersonal states [3, 4]. So, an individual’s inability to regulate or tolerate frustration contributes to the development, but especially the continuance of SUDs. Despite this well-established relationship, not much progress has been made in identifying and developing behavioral paradigms of frustration in animals within the last 50 years.

Studies assessing frustration in rodents often do so indirectly, usually *assuming* the induction of a frustration state via commonplace practices such as total or partial reward omission and quantifying motivation-related target behaviors secondary to frustration [5, 6]. It is presumed that any differences in target behaviors are due to frustration, and likewise, that lack of differences in target behaviors indicate unaffected frustration; however, in those cases, frustration itself is not quantified. Consequently, the extent to which frustration and motivation are intertwined, both behaviorally and in circuitry, remain elusive because only motivation is quantified. The current gold standard for assessing frustration behavior more directly in the form of successive negative contrast (SNC) assays, quantify frustration-related target behaviors during unexpected downshift such as latency to reward on a runway task (instrumental SNC; iSNC]) and reward intake (consummatory SNC; cSNC) [7, 8]. While quite useful, these studies lack the temporal resolution of operant bar pressing tasks; thus, measuring frustration during well-studied operant paradigms will allow the integration of frustration with motivation aspects in a single experiment in real time.

Our laboratory has proposed a novel way to study frustration-related behavior while *simultaneously* exploring motivation by simply recording bar press (BP) durations. In exploratory studies, we have shown that rats exhibit increased BP durations during frustrating conditions such as progressive ratio (PR) and extinction (EXT), where achieving a reinforcement is made more difficult or impossible, respectively [9]. This phenomenon, which will be referred to as the operant frustration effect, presents in rats self-administering drug and natural rewards alike—including cocaine, fentanyl, and sucrose pellets. In another study, we demonstrated that increased frustration for sucrose, as proxied by BP durations, predicts early “breaking” under PR in subsequent fentanyl self-administration [10]. This finding provides quantitative evidence that intrinsic frustration is a determinant of motivation for drug and was the first time to our knowledge that individual differences in frustration-related behavior have been directly linked to differential drug-taking using operant animal models. This same study also provided evidence that BP durations are characteristic of the individual animal and stable across days with similar conditions [10].

Though these findings provide compelling evidence that BP durations are a meaningful behavioral measure of frustration, the question of whether dynamic changes in BP durations are only a reflection of increased performance requirements was a major concern meriting further investigation. To this end, we have developed an operant successive negative contrast (oSNC) task which is conceptually anchored to the work of Elliot, Crespi, Amsel, Capaldi and Flaherty among many others that contributed to the theoretical and practical development of incentive downshift tasks [7, 11–14]. The oSNC allows us to investigate the effect of unexpectedly downshifting reward size in self-administering rats on BP durations. The findings from this study demonstrate that violation of expectancy, rather than increased fatigue, determine BP durations. We explore this idea further by observing decreased BP durations when expectation is violated using a frustration relief task, which together reveal bidirectional manipulation of BP durations dependent upon context.

Finally, to support the widespread implementation of this measure as a behavioral tool, we evaluate the consistency of performance variables such as replicability, robustness, and sex equitability. Overall, we conclude that BP durations are highly sensitive to frustration context, and that the operant frustration effect is replicable, robust, and present in male and female subjects alike.

## Methods

### Animals

**Fig 1:**
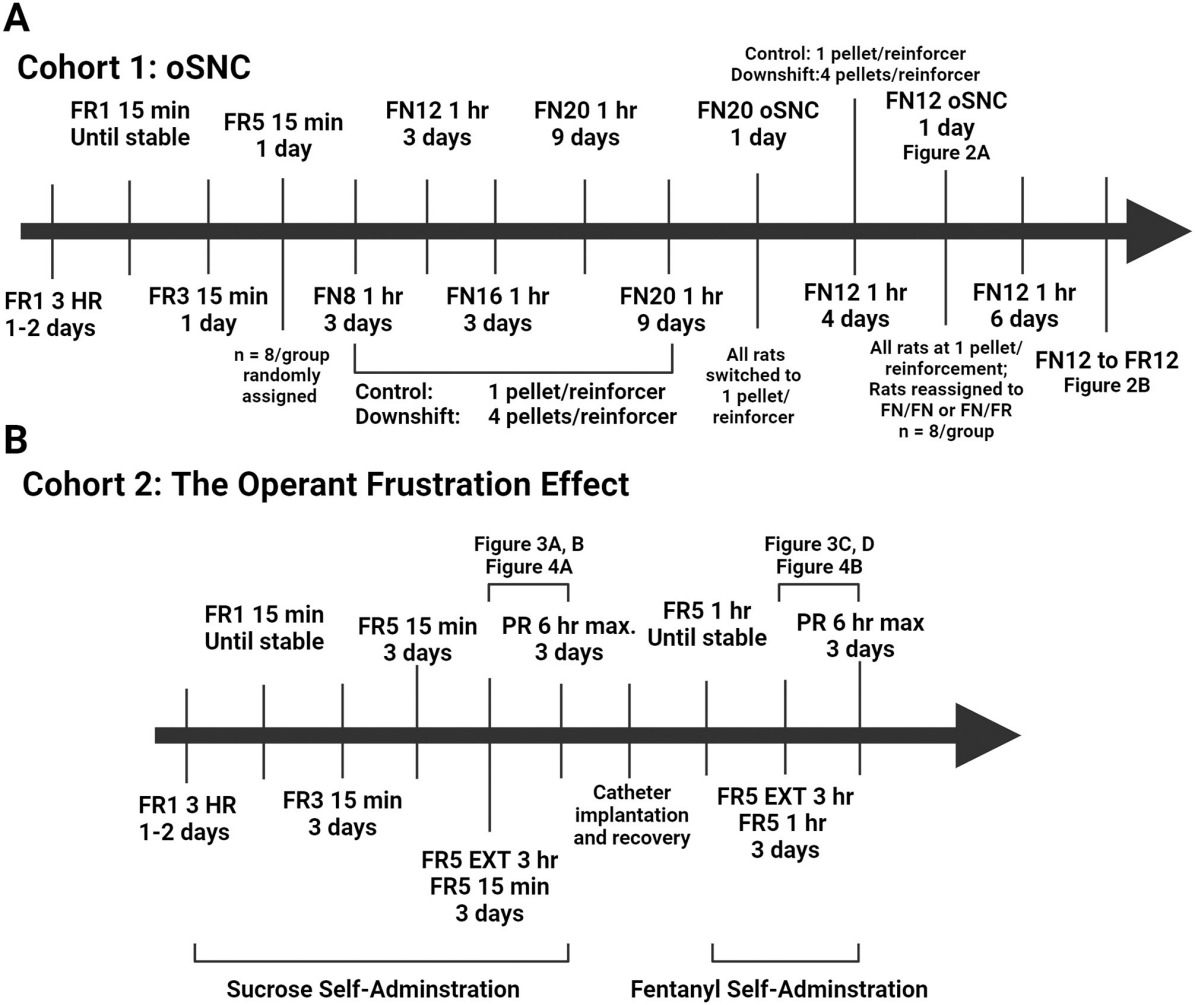
Experimental timeline for animal cohorts 1 and 2. (A) Behavioral timeline for the operant successive negative contrast experiment. (B) Behavioral timelines for the assessment of replicability, robustness, and sex equitability of the operant frustration effect, as well as relief from frustration experiments.

Sprague-Dawley rats were obtained from Envigo (Houston, TX) at 225-250g for males and 150g-175g for females; age matched 7–8 weeks. Rats were housed in an AAALAC-approved facility and conformed to the NIH Guide for the Care and Treatment of Laboratory Animals [15]. Procedures were approved by the University of Texas Medical Branch’s Institutional Animal Care and Use Committee (IACUC). Data from multiple cohorts were analyzed (Table 1). The experimental timeline for the operant successive negative contrast and the operant frustration effect is outlined in Fig 1A and 1B, respectively, for clarity.

**Table 1:** Organization of experimental cohorts.

Experiment (s)	Cohort used
Operant Successive Negative Contrast	1
Replicating Operant Frustration Effect, Relief From Frustration, Sex Equitability, Robustness	2
WSDR Experiment 1	3
WSDR Experiment 2	4
WSDR Experiment 3	5
WSDR Experiment 4a, 4b, 4c	6
WSDR Experiment 5	7

Details of cohorts used for each experiment.

### Sucrose training and self-administration

Rats were food regulated to 85% ad libitum body weight, and then trained to press a bar for banana-flavored sucrose pellets (45 mg, Bio-Serv). To learn the operant response, rats underwent one 120 minute fixed ratio (FR) session at FR1 (one press yields one reinforcement and cue light activation) where a non-contingent pellet was provided every 10 minutes to incentivize exploration. The following days, rats underwent a daily FR session, starting at FR1 until stable pressing was achieved. Subsequent reinforcement programs are specific to each experiment. Higher fixed ratio contingencies require 3 (FR3), 5 (FR5), or 12 (FR12) responses before a reinforcement is administered and cue lights are activated. Unless otherwise stated, sucrose FR sessions were 15 minutes in length.

### Transition to drug self-administration

Unless otherwise stated, rats were returned to *ad-libitum* feeding upon completion of sucrose schedules, 7 days after which rats were anesthetized with ketamine (100 mg/kg IP) and xylazine (10 mg/kg, IP) and implanted with indwelling intrajugular silastic catheters as described previously [16, 17]. To maintain catheter patency, catheters were flushed daily with 0.1 ml of heparinized (10 U/ml) saline with ticarcillin (0.067g/ml). Following a 7 day recovery period from catheter surgery, animals were placed in operant chambers to self-administer either cocaine HCl (0.5 mg/kg/infusion) or fentanyl HCl (32 μg/kg/infusion), both from the NIDA Drug Supply Program. Subsequent reinforcement programs are specific to the section of the study.

### Statistics

Data containing more than two observations were analyzed by repeated measures one-way ANOVA, repeated measures two-way ANOVA, or a mixed-model analyses in the case of missing values. Geisser-Greenhouse corrections were employed when the sphericity assumption was violated. Post-hoc analyses used Tukey’s multiple comparisons tests. Data containing two observations were evaluated using independent or paired student’s T-tests, as appropriate. Welch’s corrections were used for T-tests that violated the sphericity assumption. For WSDR experiments, only comparisons to the initial dose are presented for simplicity. Moreover, some WSDR data violated the assumption for normality, which were analyzed using non-parametric tests and post-hoc analyses. All statistics were performed using GraphPad 9 software. Rats that obtained fewer than 4 reinforcements for any given experiment were not considered in that analysis; one control animal in the operant successive negative contrast experiment was excluded from analysis for this reason.

### Operant successive negative contrast

#### Training

A total of 16 male rats were trained to press for sucrose pellets as described in the Sucrose training and self-administration section. This preceded one session of FR3 and one session of FR5. For the rest of the training sessions, rats were randomly assigned into groups self-administering 1 or 4 pellets per reinforcement (n = 8 per group). Rats were trained on the frustrative nonreward (FN) procedure detailed in [10], starting with FN8 (i.e., 8 presses per reinforcer). Briefly, the FN task makes use of the house light and cue lights above each lever to incrementally signal points: the trial begins with all 3 lights on, and one cue light is turned off after each quarter of the contingency requirement is achieved. For FN8, a cue light is turned off every two presses. The final two presses result in a reinforcement being delivered and all lights illuminated, signaling the start of the next trial. Higher FN contingencies require more presses per incremental step (e.g., FN 12 is 3 presses per cue, etc). Rats underwent a daily 60 minute FN session where contingency was increased after 3 sessions according to the following progression: FN8, FN12, FN16. The subsequent 9 sessions were at FN20.

#### oSNC

For the negative contrast session, the group originally receiving 4 pellets per reinforcer was provided 1 pellet per reinforcer and the control group remained unchanged at 1 pellet per reinforcement. Responses contributing to the first reinforcer were not analyzed because rats are unaware of the downshift until they receive the first reinforcer. A negative contrast was first attempted under FN20, but rats were unable to achieve consistent responding with both groups averaging fewer than 5 reinforcements per session. Subsequently, rats were placed back in their original 4-pellet and 1-pellet groups and dropped from FN20 to FN12 to increase the average number of reinforcements obtained by each animal. After 4 sessions on FN 12, the negative contrast was re-examined, and all rats received 1 pellet per reinforcement for the next 6 sessions. To mitigate individual differences in baseline durations, data across groups were analyzed as a frustration index (i.e., change score) by dividing the value of durations during the negative contrast session over the average of the final 2 sessions before changing the reinforcer size. A score greater than 1 on the frustration index represents durations that increased compared to baseline levels. This approach is taken from previously published literature [10]. One rat in the control group was excluded due to insufficient bar pressing (fewer than 4 reinforcements).

#### Altering cue protocol: FN vs FR

It has been shown that cue lights serve as secondary reinforcers [18], thus it is important to explore whether any unexpected change in schedule not related to frustration could also yield effects on BP durations. To this end, rats were randomly reassigned into FN 12 (n = 8) or a typical FR12 (one cue after all 12 presses; n = 8) groups, counterbalanced by previous oSNC experience. With this shift, amount of labor required for each reinforcement was unchanged within and between groups, but an alternative cue protocol was employed between groups.

### Relief from frustration

To assess relief from frustration in sucrose, rats underwent a period of cued reward omission immediately followed by a period of normal reward availability. For sucrose-pressing rats, a 4 hr FR5 cued extinction (EXT) phase was immediately followed by a 15 minute, normally reinforced FR5 phase. For fentanyl-pressing rats, a 2 hr FR5 cued EXT phase was immediately followed by a 1 hr normally reinforced FR5 phase. The average BP durations for the EXT and relief phases were compared in male and female rats self-administering sucrose or fentanyl. Rats earning fewer than 3 reinforcements during the relief phase were excluded from the analysis due to failure to reinstate responding. In total, 3 male and 4 female rats were excluded from both the sucrose and fentanyl analyses for this reason.

### Determining sex equitability

Our previous findings were obtained using male rats only [9]. Hence, in this investigation, we measured BP durations during FR5, EXT, and PR in male and female rats to both replicate the operant frustration effect in males and explore sex as a biological performance variable. Ten rats of each sex (N = 20 total) were tested for sucrose self-administration and subsequent fentanyl self-administration.

Sucrose: After 4 sessions of stable FR1 responding, rats underwent a daily session according to the following progression: FR3 (3 sessions), FR5 (3 sessions), cued FR5 EXT (3 sessions), and PR (3 sessions). EXT sessions were 4 hrs and rats were provided cues after every 5 bar presses as usual with sucrose reward omission. EXT sessions were immediately followed by a normal FR5 session to prevent extinction from affecting the next session. For PR, each successive reinforcement requires an increasing number of bar presses according to a semi-logarithmic progression (i.e. 1, 2, 4, 6, 9, 12, 15, 20…) [19] Richardson & Roberts, 1996). “Breaking” during PR sessions (including drug) was defined as 1 hr without obtaining a reinforcement, with a maximum session length of 6 hrs. “Break point” is defined as the last contingency requirement achieved before “breaking”. In these experiments, all rats met the criteria for classic “breaking”.

Fentanyl: Rats transitioned to drug self-administration as described in the Transition to drug self-administration section. After 4 sessions of stable FR1 fentanyl self-administration (38 μg/kg/inf), rats underwent a daily session according to the following progression: FR3 (4 sessions), FR5 (4 sessions), FR5 EXT (3 sessions), and PR (3 sessions). EXT sessions were 3 hrs and normal cues were provided every 5 presses. EXT sessions were immediately followed by a maintenance FR5 session (60 min) to prevent withdrawal. Two female rats were removed from the study due to catheter issues. One female rat did not complete extinction or progressive ratio sessions. In addition, rats that did not complete a given session were removed from the analysis for this segment. Another female and three male rats were removed from the progressive ratio analysis for this reason.

### Assessing robustness

Though we have previously identified robust increases in BP durations across three different reinforcers [9], the extent of robustness, individual differences, and sex differences, have not been previously presented. In the current manuscript, we further analyzed our male and female data for both sucrose and fentanyl self-administration. Individual average durations for the FR5 maintenance schedule were plotted against the average durations for EXT or PR schedules. The scatterplots of individual data points are shown in relation to a 1-to-1 ratio, indicating parity of FR5 durations to either EXT or PR durations. Data points falling above the 1-to-1 threshold indicate increased BP durations compared to the FR5 maintenance schedule and vice versa. Hence, this analysis demonstrates which schedules result in longer BP durations at the individual subject level as a demonstration of robustness and individual differences.

### Within-session dose-response

Our previous research addressed dose dependency of BP durations, but due to the loading effect seen with cocaine at the beginning of each session and the limited dose range studied were unable to resolve increases in BP durations with decreasing dose [9]. To better investigate the possibility of dose-responsivity of BP durations, and the replicability of this effect, we analyzed WSDR data from control animals from multiple different studies exploring the effect of different viral vectors on cocaine and fentanyl self-administration behavior. Moreover, we extend our WSDR findings by altering the time interval between each dose downshift and expanding the dose range in cocaine or fentanyl self-administering rats. Data were obtained from multiple animal cohorts (Table 1) used for assessing the effect of genetic (WSDR Experiments 1–3, 5) or chemogenetic (WSDR Experiment 4) manipulations on operant self-administration. In WSDR Experiments 1–3 and 5, only data from animals injected with 1 μL of an adeno-associated *control* viral vector expressing GFP and a *non-targeted* short hairpin RNA were used. This control vector has not previously affected self-administration behavior [20]. In WSDR Experiment 4, data were collected under control conditions (i.e., no activation of the receptor).

#### WSDR Experiment 1: Cocaine

Rats were trained and tested on sucrose self-administration and transitioned to self-administer cocaine as described above. Rats had maintenance responding and extinction training prior to 3 sessions of within-session dose-response (WSDR), which began with a full dose of 0.5 mg/kg/infusion that was halved every 30 minutes for the duration of the session (4 hrs). Data from the first dose were not used because rats increase BP durations during the early “loading” phase of the session [9]. Thus, only doses 0.25 mg/kg/inf and below were analyzed. BP durations were averaged for each dose over the 3 days of WSDR and were used as a measure of frustration-related behavior. Data were collected from control rats in an unpublished study analyzing the effects of an shRNA vector on cocaine operant self-administration. The data analyzed are from the 10 rats that were injected bilaterally into the nucleus accumbens shell (shNAc) with 1 μL of adeno-associated *control* viral vector expressing GFP and a non-targeted short hairpin RNA [16, 17, 21, 22]. One rat was excluded from the analysis due to a compromised catheter.

#### WSDR Experiment 2: Cocaine

As a replication of WSDR Experiment 1, WSDR Cocaine Experiment 2 was performed in a separate set of rats tested at a different time. Data were collected from control rats in an unpublished study analyzing the effects of an shRNA vector on cocaine operant self-administration. The data analyzed are from the 10 rats that were injected bilaterally into the nucleus accumbens shell (shNAc) with 1 μL of adeno-associated *control* viral vector expressing GFP and a non-targeted short hairpin RNA [16, 17, 21, 22].

#### WSDR Experiment 3: Fentanyl

Data were analyzed from a different unpublished study of the effects of another shRNA vector on fentanyl operant self-administration. The data analyzed are from 10 rats that were injected bilaterally into the shNAc with the control viral vector, as previously described, but prior to sucrose self-administration. Unlike the cocaine studies, the first dose of this study *was* analyzed because fentanyl self-administration does not have the same loading effect as cocaine self-administration on BP durations [23]. With a starting dose of 38 μg/kg/inf, doses were halved every 30 min. Three rats were dropped earlier in the study due to compromised catheters.

#### WSDR Experiments 4a-c: Cocaine extension

The following three experiments were conducted with an expanded dose range (12 doses) in one set of rats. After the first dose, the dose was decreased every 10, 15, or 30 minutes in Experiments 4a, 4b, and 4c, respectively. These data were collected from an unpublished study analyzing the effects of a chemogenetic vector on cocaine self-administration. The data analyzed are from rats that were injected bilaterally with the DREADD (hM4D, Gi) viral vector into the shNAc (n = 10 per cohort). The data were collected under control conditions (i.e., no activation of the receptor). The vector injections occurred simultaneously with catheter implantation. Instead of halving doses at intervals, doses progressed as follows: 358, 358, 201, 113, 64, 36, 20, 11, 6.4, 3.6, 2, and 1.1 μg/kg/inf. The first dose was not analyzed due to the cocaine loading effect. One rat was dropped from all three parts due to unstable responding. One additional rat was removed from analysis due to missing values in the last two doses of Experiment 4b.

#### WSDR Experiment 5: Fentanyl extension

The following experiment was also conducted with an expanded dose range (12 doses). Doses were halved every 20 minutes. Data were collected from control rats of an unpublished study analyzing the effects of another shRNA vector on fentanyl operant self-administration. The data analyzed are from 6 rats that were injected bilaterally into the shNAc with the *control* viral vector, as previously described. In addition, these rats underwent a daily period of acute enrichment (30 min) prior to each self-administration session. The first dose of this study was analyzed because fentanyl self-administration does not have the same loading effect as cocaine self-administration on BP durations [9]. Due to extinction in the lowest doses, only the first 10 doses were analyzed.

## Results

### Dynamic changes in BP durations are based on reward expectation

#### Operant successive negative contrast produces increased BP durations

**Fig 2:**
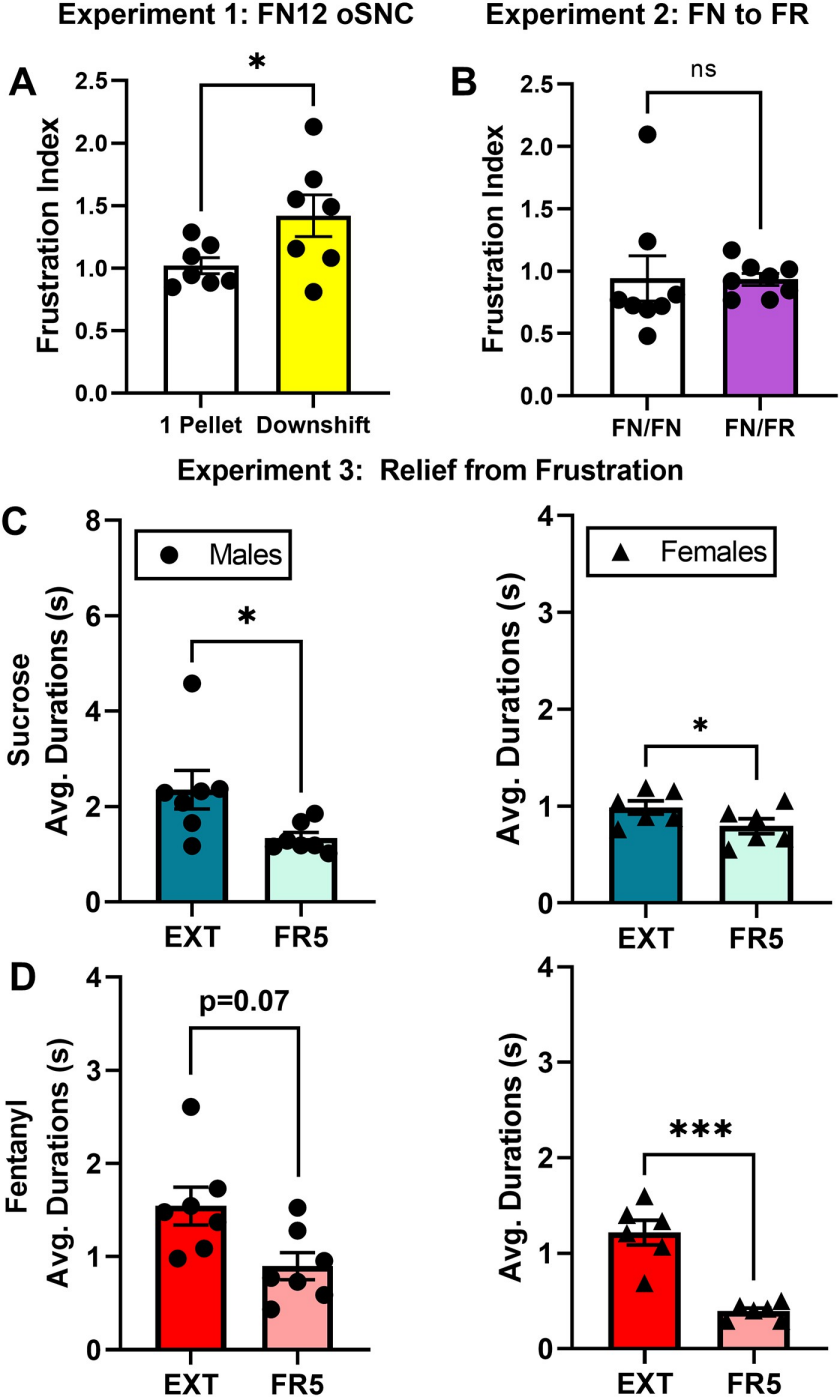
Changes in BP durations are due to psychological factors. (A) Frustration indices (mean ± SEM) for control and incentive downshift groups under the FN12 schedule during the downshift session. Frustration index is average BP duration during test session divided by average of the previous two sessions. (B) Frustration indices of rats switched from FN12 to the FR12 schedule (FN/FR) compared to rats that remained on FN schedule (FN/FN). (C) Average BP durations during cued EXT phase and the FR5 phase immediately afterwards in male (left) and female (right) rats self-administering sucrose pellets. (D) Average BP durations during cued EXT phase and the FR5 phase immediately afterwards in male (left) and female (right) rats self-administering fentanyl. Bars are mean ± SEM. Individual male data represented by circles; individual female data represented by triangles. *P < 0.05, ***P < 0.001.

After rats were trained to obtain 1 or 4 pellets per reinforcement on an FN12 task, we did not detect any differences across groups in BP durations at baseline (*t*(14) = 1.34, *p* = 0.20). When the 4-pellet group was downshifted to 1 pellet such that both groups were pressing for the same reward size, only the downshifted group (*t*(7) = 2.81, *p* = 0.02), and not the control group (*t*(6) = 0.81, *p* = 0.42), increased BP durations compared to the average of the two prior sessions, confirming that a negative contrast was achieved. Across groups, the downshifted group exhibited significantly greater frustration indices when compared to control rats (*t*(13) = 2.20, *p* = 0.04; Fig 2A). These results indicate that increases in BP durations are due to the violation of reward expectation in the downshifted group.

The mean number of reinforcements obtained during each session was lower in the 4 pellet group prior to downshift compared to controls (*t*(13) = 2.25, *p* = 0.04), though the mean total number of pellets obtained was still greater in the 4 pellet group. Upon downshift, there was no difference across groups in number of reinforcements obtained (*t*(13) = 1.17, *p* = 0.25).

#### Altering cue protocol does not affect BP durations

Prior to the changing the cue protocol from FN12 to FR12, there were no significant differences between groups in BP durations at baseline (*t*(14) = 0.21, *p* = 0.83). After six sessions of all rats pressing for 1 pellet on an FN12 schedule, half of the rats were placed on an FR12 schedule. We found that within groups, neither group increased their BP durations significantly during the switch (Control, *t*(7) = 1.66, *p* = 0.13; FN to FR, *t*(7) = 0.97, *p* = 0.36). According to a Welch-corrected unpaired t-test, frustration indices across groups did not significantly differ from each other (*t*(8) = 0.04, *p* = 0.97, Fig 2B) or number of reinforcements obtained (*t*(12.84) = 1.41, *p* = 0.18).

#### Relief from frustration results in lower BP durations

Both male and female rats self-administering sucrose (Males, *t*(6) = 2.45, *p* = 0.049; Females t(5) = 2.99, p = 0.03) or fentanyl (Males, *t*(6) = 2.16, *p* = 0.07; Females, *t*(5) = 7.11, *p* = 0.0008) exhibited decreased BP durations in the relief phase compared to the preceding EXT phase (Fig 2C and 2D).

### The operant frustration effect is replicable, sex equitable, and robust

**Fig 3:**
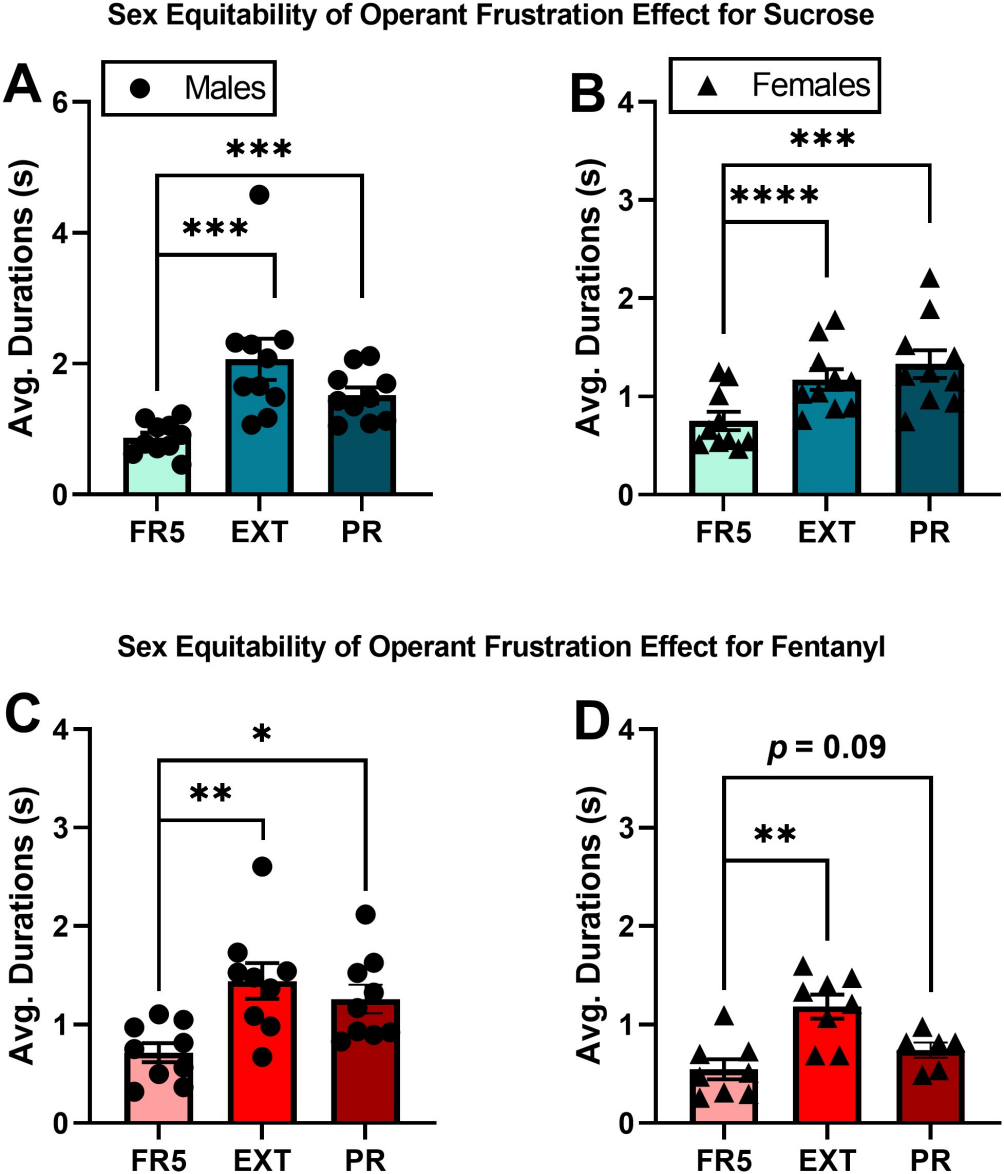
The operant frustration effect is present in male and female rats. (A) Average BP durations for male (left) and female (right) rats self-administering sucrose during EXT and PR. (B) Average BP durations for male (left) and female (right) rats self-administering fentanyl during EXT and PR. In A and B, EXT and PR are each compared to FR5. Bars are mean ± SEM. Individual male data represented by circles; individual female data represented by triangles. *P < 0.05, **P < 0.01, ***P < 0.001, ****P < 0.0001.

**Fig 4:**
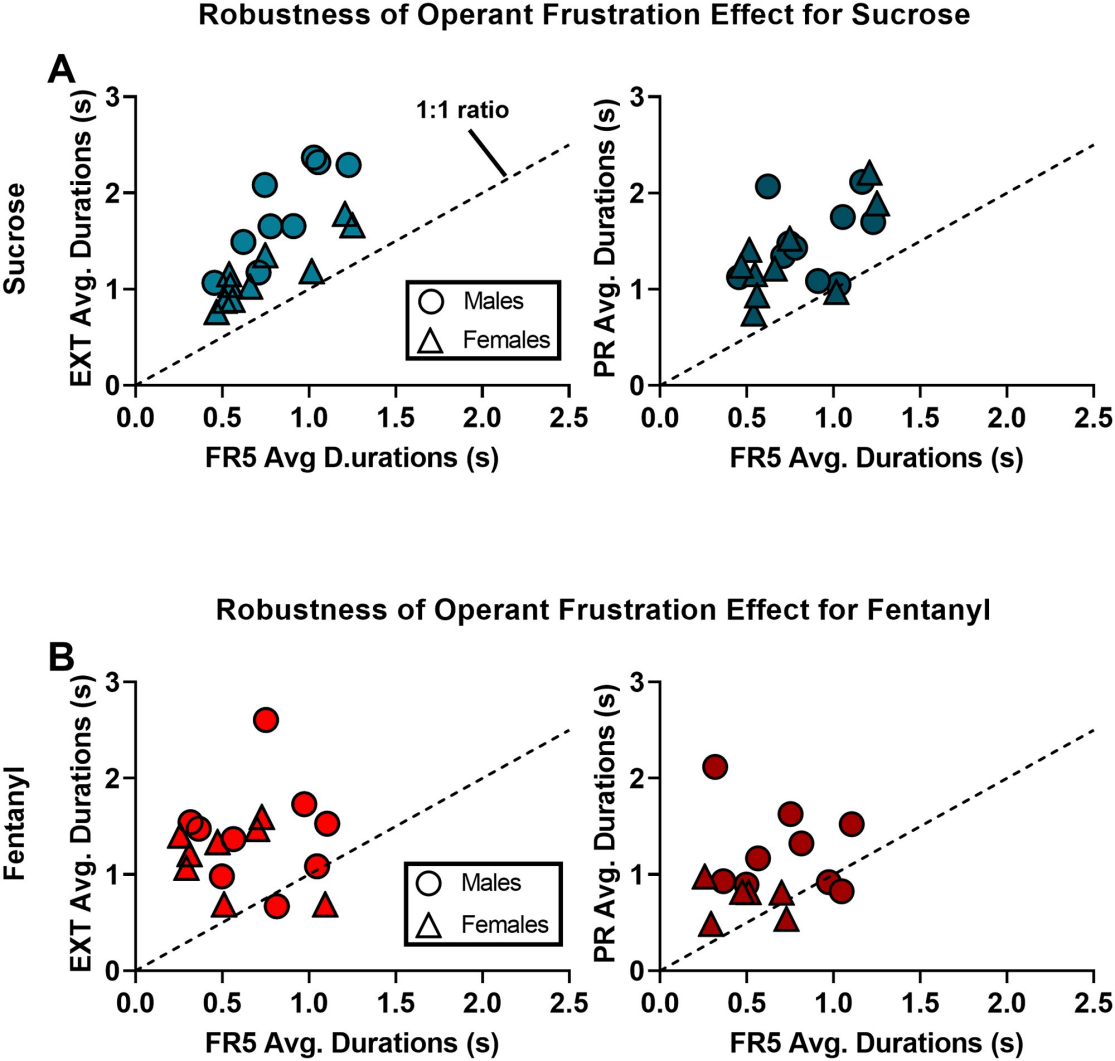
Operant frustration effect is robust. (A) Scatter plots of mean BP duration for individual rats self-administering sucrose during EXT (left) and PR (right). (B) Scatter plots of mean BP duration for individual rats self-administering fentanyl during EXT (left) and PR (right). Dashed line represents parity for FR5 vs. EXT (left) or FR5 vs. PR (right). Individual male data represented by circles; individual female data represented by triangles.

Both male and female rats increased mean BP durations under extinction from sucrose (Males, *t*(9) = 4.53, *p* = 0.001; Females *t*(9) = 9.12, *p* < 0.0001) as well as fentanyl (Males, *t*(8) = 3.53, *p* = 0.007; Females, *t*(7) = 3.58, *p* = 0.009) compared to their previous FR5 sessions (Fig 3A). These results replicate our original findings in male rats and moreover support BP durations as a measure of frustration equally applicable in both male and female rats self-administering sucrose or fentanyl under complete reward omission. Except for one male and one female rat self-administering fentanyl, all rats self-administering sucrose or fentanyl exhibited longer BP durations during extinction compared to their previous FR5 sessions, demonstrating excellent robustness among both sexes (Fig 4).

Both male and female rats self-administering sucrose exhibited higher average BP durations for PR compared to the previous FR5 sessions (Males, *t*(9) = 5.20, *p* = 0.0006; Females *t*(9) = 5.69, *p* = 0.0003). Except for one female rat, all rats self-administering sucrose had increased durations for PR compared to their previous FR5 sessions. Male rats self-administering fentanyl exhibited increased average BP durations during PR compared to previous FR5 sessions (*t*(8) = 2.82, *p* = 0.02), replicating our original findings in male rats. In female rats self-administering fentanyl, only a trend for increased durations during PR was detected (*t*(5) = 2.02, *p* = 0.09), likely only due to the loss of power in this group (n = 6). These findings suggest that the operant frustration effect of increased BP durations during PR is present in female rats as well as male rats (Fig 3). At the individual level, all except one female rat and two male rats self-administering fentanyl exhibited increased BP durations during PR compared to FR5, again demonstrating high robustness among both sexes (Fig 4).

### BP durations increase in a dose-dependent manner during WSDR

#### WSDR Experiments 1 and 2: Cocaine

**Fig 5:**
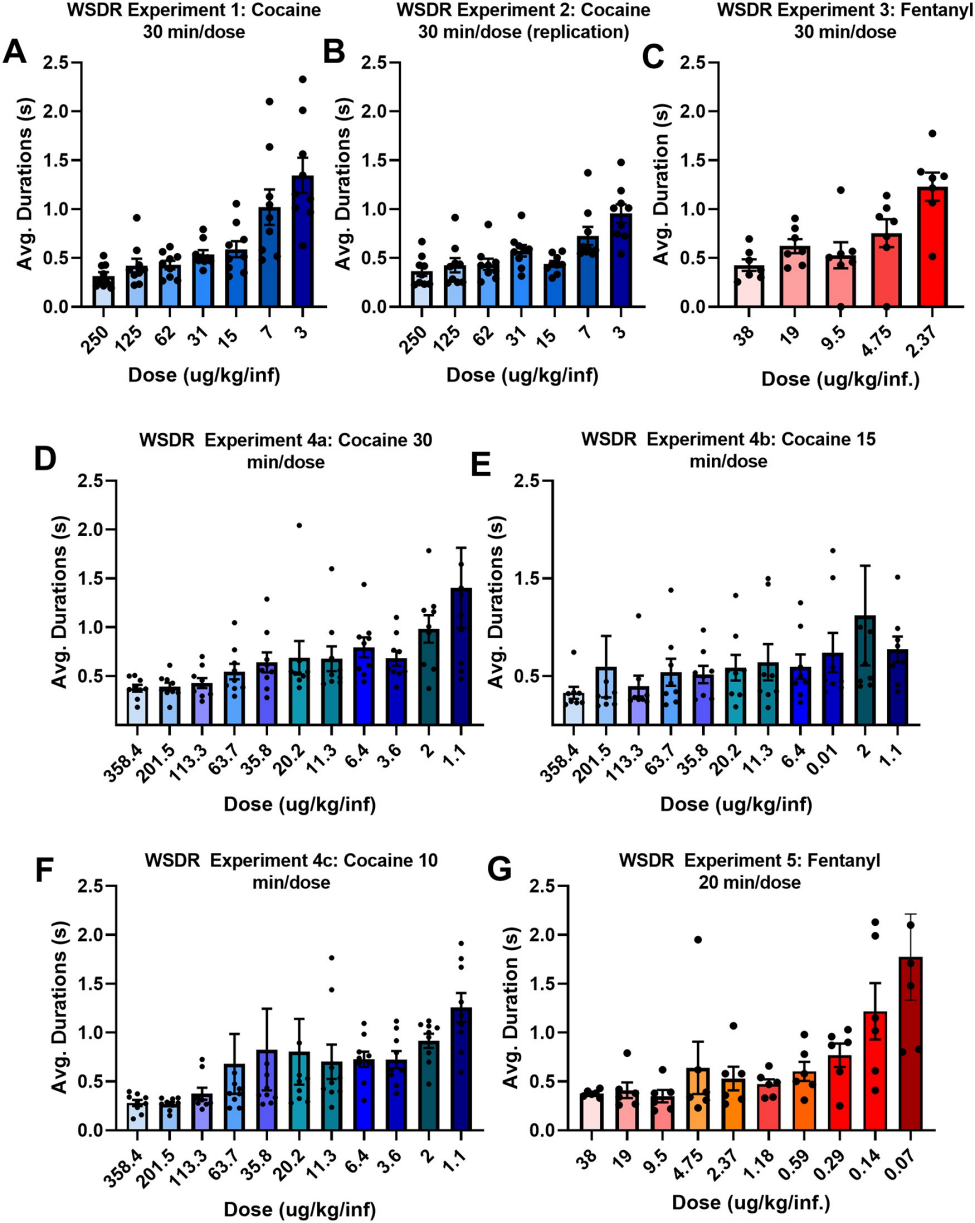
Dose dependency of BP durations. (A, B). Average BP durations for rats self-administering descending doses of cocaine during within-session dose-response (WSDR) Experiment 1 (A; 30 min/dose) and WSDR Experiment 2 (B; 30 min/dose). (C) Average BP durations for rats self-administering descending doses of fentanyl during WSDR Experiment 3 (30 min/dose). (D-F) Average BP durations for rats self-administering descending doses of cocaine during WSDR Experiment 4a (D; 30 min/dose), 4b (E; 15 min/dose), and 4c (F; 10 min/dose). G. Average BP durations for rats self-administering descending doses of fentanyl during WSDR Experiment 5 (20 min/dose). Bars are mean ± SEM. Significance is reported in the text. Significance marks are not shown due to overcrowding. Some individual data points are not shown to avoid distortion. In D, one datum in the 1.1 ug/kg/inf dose lies out of bounds. In E, one datum in the 2 ug/kg/inf and one datum in the 201.6 ug/kg/inf lie out of bounds. In F, one datum in the 20.2 ug/kg/inf, one datum in the 35.8 ug/kg/inf, and one datum in the 63.7 ug/kg/inf lie out of bounds.

For rats self-administering cocaine during Experiment 1 (Fig 5A), there was a main effect of dose on BP durations (*F*(1.35, 10.86) = 16.24, *p* = 0.001). A Tukey’s post-hoc analysis revealed that the average BP durations for four of the subsequent doses (0.03125 mg/kg/inf. *p* = 0.0012; 0.015625 mg/kg/inf. *p* = 0.013; 0.0078125 mg/kg/inf. *p* = 0.0476 and 0.00390625 mg/kg/inf. *p* = 0.004) significantly increased compared to the first dose (0.25 mg/kg/inf). Additionally, durations for the 8^th^ dose are significantly greater than all other doses. These results suggest that frustration as measured by average BP durations increases in a dose-dependent manner during cocaine self-administration.

For Experiment 2 (Fig 5B), there was a significant main effect of dose on BP durations (*F*(1.54, 10.82) = 10.91, *p* = 0.002). Post-hoc analyses revealed that average BP durations for the 8^th^ dose were significantly higher than durations for the first dose (*p* = 0.010). Moreover, there was a trend for lower durations in the first dose compared to the 7^th^ dose (*p* = 0.061). No additional differences were detected among comparisons to the first dose.

#### WSDR Experiment 3: Fentanyl

There was a significant main effect of dose on BP durations in rats self-administering fentanyl during Experiment 3 (*F*(2.03, 12.19) = 13.72, *p* = 0.001; Fig 5C). Post-hoc analyses show that the average BP durations for first dose were significantly lower than the fourth dose (*p* = 0.007) and fifth dose (*p* = 0.005). Once again, durations for smallest dose are significantly greater than all the other doses. Altogether these results demonstrate that as the doses decrease BP durations increase, suggesting frustration level is sensitive to changes in dose during fentanyl self-administration.

#### WSDR Experiments 4a-c: Cocaine extension

When dose was halved every 30 minutes, a non-parametric Friedman’s test revealed a significant main effect of dose on BP durations (*X*^*2*^(11, n = 9) = 29.92 *p* = 0.0009; Fig 5D). Multiple comparisons revealed that average BP durations for first dose were significantly lower than the durations for the last dose (*p* = 0.02). When dose was halved every 15 minutes, there was a main effect of dose on BP durations (*X*^*2*^(11, n = 8) = 27.64, *p* = 0.0021; Fig 5E). Multiple comparisons revealed that average BP durations for first dose were significantly lower than the durations for the last dose (*p* = 0.02). When dose was halved every 10 minutes, there was a main effect of dose on BP durations (*X*^*2*^(11, n = 9) = 67.37, *p* < 0.0001; Fig 5F). Multiple comparisons revealed that average BP durations for first dose were significantly lower than the durations for the ninth (*p* = 0.009), tenth (*p* = 0.04), eleventh (*p* = 0003), and twelfth dose (*p* = 0.0001). These findings replicate and expand our results from Experiment 3, suggesting that downshift intervals of 15 and 10 minutes per dose are sufficient to detect dose-dependent increases in BP durations in rats self-administering cocaine over a span of 12 doses.

#### WSDR Experiment 5: Fentanyl extension

For rats self-administering fentanyl in Experiment 5 (Fig 5G), a non-parametric Friedman’s test revealed a significant main effect of dose on BP durations (*X*^*2*^(9, n = 6) = 36.64, *p* < 0.0001). Multiple comparisons revealed that average BP durations for first dose were significantly lower than the durations for the ninth (*p* = 0.010) and tenth dose (*p* = 0.003). These findings expand our results from Experiment 3, suggesting that decreasing the downshift interval to 20 minutes per dose is sufficient to detect dose-dependent increases in BP durations in rats self-administering fentanyl over a span of 10 doses.

## Discussion

The results in this manuscript, most importantly, eliminate fatigue as a possible confounding performance factor and highlight reward expectation as a key determinant of BP durations during frustrating conditions. It is known that rodents have numerosity and that they apply counting techniques to optimize reward acquisition during operant tasks [23, 24]. “Cost” is thereby determined by the effort expenditure (i.e., number of presses) required for each reinforcement. Theoretically, then, incentive downshift should result in increased frustration-related behavior whereas a schedule change that does not affect perceived cost, such as loss of incremental cues, should not induce frustration, as supported by our findings.

First, we created an operant successive negative contrast (oSNC) task that found rats undergoing incentive downshift exhibited higher BP durations than rats always pressing for the same smaller reward. Despite equal effort expenditure per reinforcement (i.e. “cost”) in both groups, downshifted rats experience a sharp increase in the perceived cost of reward based on their expectation history, resulting in heightened frustration that can be detected by BP durations (Fig 2A). This core concept of increased perceived cost is shared across all types of SNC assays [7, 13, 25]. However, no changes in barpress durations were detected when the change in schedule (FN12 to FR12) did not change overall cost of the reinforcer (Fig 2B).

Next, we demonstrated that changes in BP durations are bidirectional and can be used to detect relief from frustration as well (Fig 2C and 2D). When rats were switched from an initially frustrated condition (i.e., EXT) to a lower cost schedule (i.e., FR5), BP durations decreased despite continued effort as the session progressed, again providing evidence that fatigue is not a key determinant of BP durations during frustration. We conclude that dynamic changes in BP durations are reflective of a psychological phenomenon based on reward expectation/perceived cost and thus can be used to study frustration in rats.

In the next experiment, we sought to investigate performance aspects such as sex differences, replicability, and robustness of the operant frustration effect. Our data show high fidelity to our original findings [9]. First, we replicated the operant frustration effect for both EXT and PR in male rats self-administering sucrose as well as fentanyl (Fig 3A and 3C) while at the same time extending the effect for female rats (Fig 3B and 3D). For robustness, we found that over 90% of rats had higher BP durations during frustrating conditions compared to baseline FR5 responding, regardless of sex, schedule (i.e., EXT vs. PR), or reward (i.e., sucrose, fentanyl). The high consistency in performance across these three factors support BP duration measurements as a useful behavioral tool for the study of frustration. Interestingly, despite robust increases in group BP durations, there exists a significant amount of individual variation within groups to make possible the study of individual differences in frustration-related behavior.

Our consistent results in both sexes is in line with existing research exploring sex differences of frustration behavior in humans, which though scarce, reveals few differences in how men and women experience frustration. In cases where differences were detected, these usually relate to the way frustration is externalized (i.e. via aggression), which is greatly confounded by the patriarchal socialization of human men [26, 27].

Finally, we extensively evaluated the dose-dependency of BP durations during dose-response paradigms. In our original publication we were unable to report a significant dose-dependent increase in bar-press durations in rats self-administering cocaine across 5 doses [9]. However, we now know that for cocaine self-administration rats exhibit a loading effect where BP durations are higher during loading, an effect that washed out the effect in our prior 5-dose study. Eliminating the first dose results in significant responsiveness to dose. In these studies, we replicated our original findings in rats self-administering cocaine twice, excluding the loading dose from analysis. Our data showed clear progressive increments in BP durations with decreasing doses of cocaine (Fig 5A and 5B). We also expanded our findings by expanding the dose range and reducing the dose interval from 30 min/dose to 15 and 10 min/dose, all of which yielded similar findings of progressively increased durations with increased frustration (Fig 5D–5F). Similar results were obtained for fentanyl self-administration except that the dose range included two low doses that produced full extinction in many rats, restricting our statistical analysis to 10 doses.

For WSDR studies, though unlikely, drug-based effects may confound our findings of increased press durations with subsequent dose downshift. Both cocaine and fentanyl have been demonstrated to induce hyperlocomotion in rats [28, 29]. However, hyperlocomotion would be expected to decrease BP durations instead of increasing them. Alternatively, drug-induced fatigue would be expected to increase duration of pressing. Despite this possibility, we are confident that this is not a major contributor to our WSDR findings for a few reasons. Firstly, the operant frustration effect was evident for both sucrose and drug self-administration. If drug-induced hyper- or hypo-locomotion were a major determinant, we would expect different results for sucrose vs. drug experiments which was not the case. Moreover, fatigue and other performance factors were ruled out as major determinants for changes in BP durations by both oSNC and relief from frustration experiments. Relief from frustration experiments also obtained equivalent results for sucrose and drug self-administration, again suggesting that drug-induced motoric effects are not a major determinant of our results.

It is quite interesting that a loading effect on BP durations is present and has been consistently replicated for cocaine and not fentanyl self-administration. Cocaine is a pure stimulant while fentanyl has both stimulant and sedative properties, which is one possible explanation for this phenomenon [29–32]. Furthermore, we cannot claim equivalence in the cocaine and fentanyl doses used for self-administration. It is plausible that using higher or lower doses of fentanyl could yield different effects on BP durations for the loading phases of self-administration.

The use of BP durations as a behavioral measure during operant tasks provides a detailed window into the animal’s emotional state, specifically pertaining to frustration. Our first publication detailed the operant frustration effect in rats self-administering sucrose, cocaine, and fentanyl [9]. We can evaluate frustration behavior in real time while simultaneously surveying motivational behaviors, which we have leveraged to assess individual differences in both dimensions before [10]. In the current study, we demonstrate that changes in BP durations are determined by psychological factors. Moreover, we show that BP durations are robust, sex equitable, and highly replicable, making this a promising behavioral tool. Widespread adoption of this technique has the potential to elevate our understanding of addiction, mood disorders, impulsivity, learning, and many other frustration-associated mechanisms.

## Conclusions

Based on the evidence compounded in these studies, we conclude that BP durations are a robust behavioral measure that can be used to quantify frustration behavior in male and female rats. These are sensitive to a variety of conditions (e.g., EXT, PR, incentive downshift, etc.) across numerous reinforcement types, including natural and drug rewards. We further conclude that fatigue and other physical performance factors are not significant determinants for changes in press durations, unlike psychological factors such as perceived cost.
